# Using MaxEnt modeling to analyze climate change impacts on *Pseudomonas syringae* van Hall, 1904 distribution on the global scale

**DOI:** 10.1016/j.heliyon.2024.e41017

**Published:** 2024-12-07

**Authors:** Sameh M.H. Khalaf, Monerah S.M. Alqahtani, Mohamed R.M. Ali, Ibrahim T.I. Abdelalim, Mohamed S. Hodhod

**Affiliations:** a Faculty of Biotechnology, October University for Modern Sciences & Arts (MSA University), 6th October City, 12566, Egypt; b Biology Department, Faculty of Science, King Khalid University, Abha, 61413, Saudi Arabia

**Keywords:** Climate change, Disease management, GIS, Maxent modeling, *Pseudomonas syringae*

## Abstract

*Pseudomonas syringae* is a pathogenic bacterium that poses a significant threat to global agriculture, necessitating a deeper understanding of its ecological dynamics in the context of global warming. This study investigates the current and projected future distribution of *P. syringae*, focusing on the climatic factors that influence its spread. To achieve this, we employed Maximum Entropy (MaxEnt) modeling based on Geographic Information Systems (GIS) to analyze species occurrence records alongside relevant climate data. The MaxEnt model was calibrated using 75 % of the occurrence data, with the remaining 25 % reserved for validation. The model's performance was meticulously assessed utilizing the area under the curve (AUC) and true skill statistics (TSS), resulting in an AUC score of 0.92, indicating excellent predictive capability. Our analysis identified key climatic parameters—temperature, precipitation, and humidity—that significantly affect the presence of *P. syringae*. Notably, our findings project an expansion of the bacterium's geographic range in the coming decades, with optimal conditions shifting toward the poles. This research underscores the significant influence of climate change on the distribution of *P. syringae* and provides valuable insights for developing targeted disease management strategies. The anticipated increase in bacterial infections in crops highlights the urgent need for proactive measures to mitigate these effects.

## 1 Introduction

*Pseudomonas syringae* Van Hall is a plant pathogenic bacterium that represents a significant threat to crops worldwide [1]. This Gram-negative, rod-shaped bacterium has garnered substantial attention from researchers, growers, and plant pathologists due to its ability to cause devastating diseases in various plant species [2]. Diseases caused by *P. syringae*, such as bacterial speck, bacterial canker, and bacterial blight, can result in substantial yield losses and economic impacts on crop production [3]. The biology and ecology of *P. syringae* are characterized by its remarkable adaptability and diversity. This bacterium exhibits a facultative pathogenic lifestyle, allowing it to thrive in diverse environmental niches, including plant surfaces, soil, and water [4,5]. *P. syringae* has evolved intricate mechanisms to colonize and persist on plant surfaces, such as forming biofilms and producing extracellular polysaccharides [6]. These adaptations contribute to its survival, spread, and ability to initiate infections in susceptible host plants. Pathogenicity is a crucial aspect of *P. syringae's* interactions with plants. The bacterium possesses an arsenal of virulent factors that enable it to manipulate and exploit host defenses [7]. One of the most notable virulence mechanisms employed by *P. syringae* is the type III secretion system (T3SS), which injects effector proteins directly into the host plant cells. These effectors interfere with plant immune responses, enabling the bacterium to evade detection and establish infection. Additionally, *P. syringae* produces phytotoxins and cell wall-degrading enzymes, contributing to disease development and tissue damage [8]. One of the remarkable features of *P. syringae* is its broad host range. It has the capacity to infect various plant species, including some significant economic-value crops like tomatoes, citrus, and beans [9]. However, different strains of *P. syringae* often display host specificity, causing disease only in certain plant species or cultivars [10]. Understanding the factors that determine host range and the mechanisms by which *P. syringae* interacts with specific plant hosts is essential for effective disease management strategies, especially the relation of these bacterial strains to climate [11]. The relationship between *Pseudomonas syringae* and climate is complex and multifaceted. Climate plays a significant role in shaping this plant pathogenic bacterium's biology, ecology, and epidemiology.

Temperature is a critical environmental factor influencing the survival, growth, and activity of *P. syringae*. Different strains of *P. syringae* exhibit distinct temperature ranges for optimal growth and disease development [12]. Some strains thrive in cooler temperatures, while others adapt to warmer conditions. Changes in temperature patterns, such as seasonal variations or long-term climate shifts, can affect the profusion and distribution of *P. syringae* populations. Precipitation and rainfall also play essential roles in the survival and spread of *P. syringae*. Rainfall events provide moisture necessary for bacterial growth and facilitate dispersal through splashing or water runoff [13]. High humidity levels on plant surfaces can create favorable conditions for *P. syringae* colonization and infection. Additionally, rainfall patterns can affect disease severity, as wet conditions favor the spread and persistence of bacterial populations [14].

Climate change is expected to affect the interactions between *P. syringae* and host plants significantly. Changes in the pattern of rainfall and temperature can impact the geographic distribution of *P. syringae* strains and their ability to cause disease [15]. Changes in temperature regimes can influence the timing and severity of disease outbreaks, as well as alter the phenology of both the bacterium and its host plants [16]. Warmer temperatures may extend the growing seasons and favor the proliferation of *P. syringae* populations, potentially leading to increased disease pressure. Geographical Information System GIS allows the integration and analysis of climate data from diverse sources, such as weather stations, remote sensing, and climate models, to provide a spatially explicit representation of climatic conditions [2]. By overlaying climate data with spatial information on *P. syringae* occurrences and disease incidence, GIS facilitates the identification of climate patterns associated with disease outbreaks and the characterization of climate suitability for bacterial growth and pathogenicity [17].

MaxEnt (Maximum Entropy) is a well-known modeling technique that utilizes species occurrence records and environmental variables to predict species distributions [18]. Its ability to extend species distributions beyond recorded occurrences by utilizing the link between species presence and environmental data makes it an ideal tool for assessing the implications of climate change. By incorporating climate variables, such as temperature, precipitation, and humidity, MaxEnt modeling can project the predicted distribution of *P. syringae* under different climate change scenarios. In addition to predicting the possible distribution of *P. syringae*, MaxEnt modeling can also identify the critical climatic factors driving its occurrence [19]. By examining the contribution of different climate variables in the model, researchers can gain insights into the specific climatic conditions that favor *P. syringae* growth and pathogenicity. This information can guide further research on the biology and ecology of *P. syringae* and inform the development of more precise and targeted disease management strategies.

It is essential to identify particular research gaps and develop focused queries and hypotheses. Significant gaps encompass an inadequate comprehension of the interactions between *Pseudomonas syringae* and other microbial communities, the regional variability in climate change effects, the impact of agricultural practices on the bacterium's dissemination and virulence, and the necessity for longitudinal studies on *P. syringae* populations. Future research should examine the influence of microbial interactions on pathogenicity, analyze the impact of agricultural practices on infection rates, and evaluate the effects of climate variations on the pathogen's life cycle. Furthermore, analyzing the correlation between habitat suitability changes and crop productivity may reveal regions at risk that require management interventions. The proposed hypotheses suggest that microbial populations substantially affect *P. syringae* pathogenicity, that intensive agricultural methods result in elevated infection rates, and that variations in temperature and humidity are associated with increased infections. These factors will offer an extensive comprehension of *P. syringae* dynamics within the framework of climate change.

The purpose of this research is to determine the climatological factors that influence the dynamics of *P. syringae* and to make predictions about its current and future spread based on this information. We create prediction distribution maps by combining records of species occurrence and climate data, especially environmental factors. This is accomplished via the utilization of MaxEnt modeling. Also, the aims include analyzing the suitability of different locations for the presence of *P. syringae* by analyzing bioclimatic covariates. An additional aspect that was investigated was the influence that these environmental circumstances had on the dynamics of the pathogen. Our understanding of the impact that climate change has on *P. syringae* will be enhanced as a result of the findings of this study, which will also make it easier to develop disease management measures that are specifically matched to the situation.

## 2 Material and method

### 2.1 Occurrence data collection

**Fig. 1:**
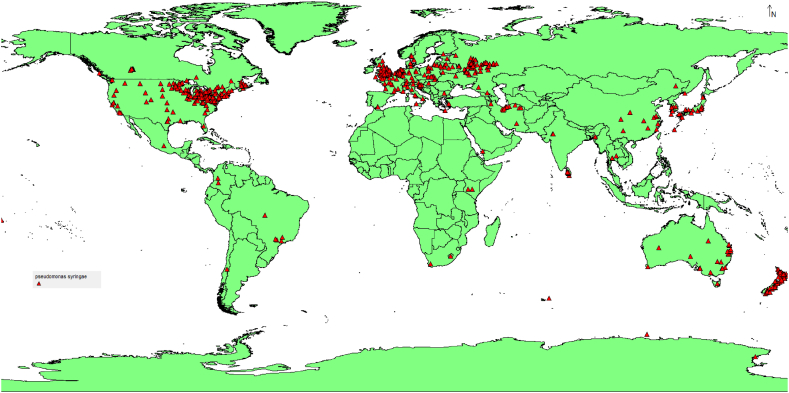
The occurrence records of *Pseudomonas syringae* used in the present study.

The occurrence data for *Pseudomonas syringae* Van Hall were obtained from internet archives such as GIBF [20]. To ensure data accuracy, duplicates were removed, resulting in 979 unique sites. These data were then transformed into a comma-delimited (CSV) format (Fig. 1) for further analysis.

### 2.2 Climatological data

The WorldClim global climate database, which has a spatial resolution of 2.5 arc-minutes or 5 km2 at the equator (accessed in December 2023), serves as the source for the extraction of nineteen bioclimatic variables (**Supplementary.1**). Converting fifteen bioclimatic variables into ASCII format was accomplished with the help of ArcGIS version 10.7. Since spatial artifacts were present, bioclimatic layers 8–9 and 18–19 were not included in the analysis [21]. To address the issue of multicollinearity, the Pearson correlation coefficient (r) was used to choose five non-correlated bioclimatic variables for further research. This was done in order to solve the issue of multicollinearity. The SDMTools in ArcGIS 10.3 (Universal tool; Remove highly correlated variables) were used in order to conduct correlation tests [22]. Bio_1, bio_5, bio_11, bio_14, and bio_17 were the variables that were being discussed here. Further, future estimates for two typical concentration paths (RCPs 2.6 and 8.5) in 2050 and 2070 were acquired from www.worldclim.org and converted into ASCII format using ArcGIS 10.7 [19]. These projections were obtained in 2050 and 2070.

### 2.3 Maxent modeling

Maxent v3.4.3e, a maximum entropy method, was employed to assess the biological niches and habitat suitability of *Pseudomonas syringae*. This method is known for its statistical power and ability to generate reliable predictive models using presence-only records [18]. To ensure model accuracy, 75 % of the occurrence records were used for training, while the remaining 25 % were used for testing [23]. As part of the modeling procedure, the maximum iteration count were limited to 1,000, and set the maximum background point count to 10,000. In order to improve the model's performance, a 5-fold cross-validation was carried out [24].

### 2.4 Model evaluation

The Maxent model, utilizing the five selected bioclimatic variables and 979 presence-only locations, was run to predict the potential habitat range of *Pseudomonas syringae*. Two independent groups of occurrence records were created, with 75 % used for model training and 25 % for testing [25,26]. The evaluation of the model's performance was conducted through the area under the curve (AUC), with values exceeding 0.9 signifying excellent performance [27]. The jackknife test was utilized to determine the key bioclimatic factors affecting the distribution of the species [28]. Furthermore, true skill statistics (TSS) were employed to evaluate the precision of the projected models. TSS values span from −1 to 1, where positive values nearing 1 suggest a robust correlation between the projected model and the distribution of the species [29].

## 3 Results

### 3.1 Model evaluation

**Fig. 2:**
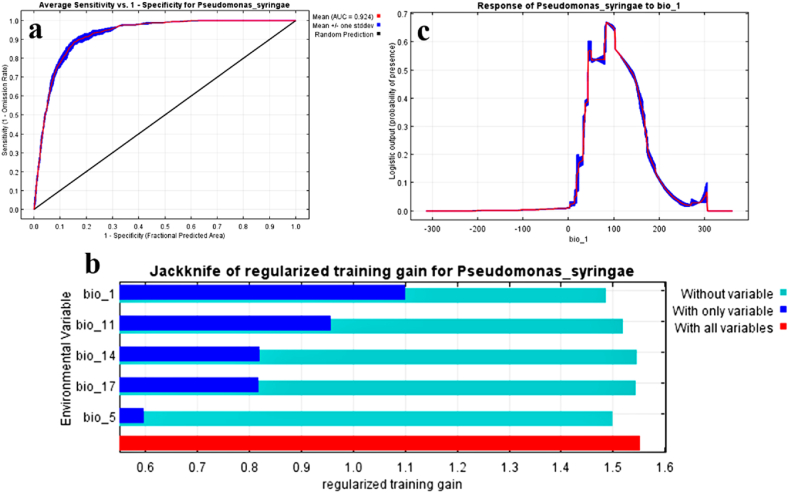
**a.** The graph presents the receiver operating characteristic (ROC) curve for the *Pseudomonas syringae* dataset, averaged over multiple replicate runs. Specificity is determined based on the expected area rather than the actual commission, with an AUC value of 0.92. **b** displays the Jackknife results for the primary contributing variable, while panel c illustrates the response curve for the most impactful bioclimatic factor (bio_1).

To evaluate the effectiveness and reliability of our MaxEnt model, we employed the True Skill Statistic (TSS) and the Area Under the Curve (AUC) metrics. The AUC score obtained was 0.92, reflecting outstanding model performance in analyzing the distribution of this bacterium (see Fig. 2a). Additionally, a TSS value of 0.75 confirmed the model's accuracy. To assess the influence of various environmental factors on the bacterium's distribution, we performed a Jackknife test focused on the five most significant bioclimatic variables. These included bio_1 (Annual Mean Temperature), bio_5 (Maximum Temperature of the Warmest Month), bio_11 (Mean Temperature of the Coldest Quarter), bio_14 (Precipitation of the Driest Month), and bio_17 (Precipitation of the Driest Quarter) (Fig. 2b). The Jackknife test results indicated that bio_1 (Annual Mean Temperature) contributed the most, with a percentage of 38.3 %, highlighting its critical role in the bacterium's distribution. Following bio_1, the contributions were 21 % for bio_17, 18.8 % for bio_14, 12.2 % for bio_11, and 9.7 % for bio_5.Moreover, the response curve analysis revealed that the optimal temperature range for the bacterium lies between 10 °C and 15 °C (as shown in Fig. 2b). This finding underscores the significance of bio_1 (Annual Mean Temperature) in shaping the distribution patterns of this bacterium (Fig. 2c).

### 3.2 Current habitat suitability

**Fig. 3:**
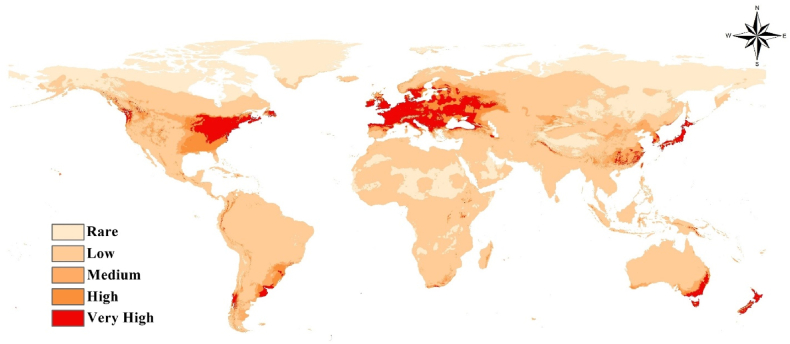
Current prediction of *Pseudomonas syringae* distribution.

The current habitat suitability modeling of *Pseudomonas syringae* reveals interesting patterns across different regions of the world (Fig. 3). The analysis indicates that this pathogenic bacterium exhibits high suitability in several areas, including Europe, the eastern part of North America, Japan, the eastern part of China, the eastern part of Australia, and New Zealand. On the other hand, Africa generally shows low habitat suitability for *Pseudomonas syringae*, except for the southern part of South Africa. Furthermore, the low habitat suitability observed in Africa, apart from the southern part of South Africa, which shows high and very high habitat suitability, highlights the potential differences in environmental factors that may limit the establishment and survival of *Pseudomonas syringae* in this continent. South America, on the other hand, shows low habitat suitability except along the Andes and parts of Argentinian coasts on the Atlantic.

### 3.3 Evaluation of the risk of invasion of *P.syringae* to new areas under changing climate

**Fig. 4:**
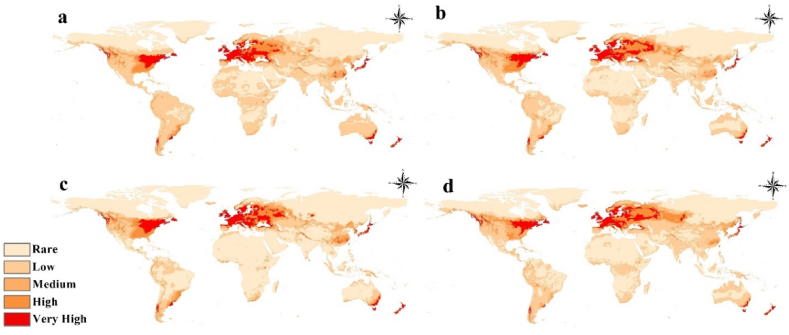
Future distribution projections for *Pseudomonas syringae* worldwide under two representative concentration pathways: **a.** 2050 for 2.6; **b.** 2050 for 8.5; **c.** 2070 for 2.6; and d. 2070 for 8.5 were made.

**Fig. 5:**
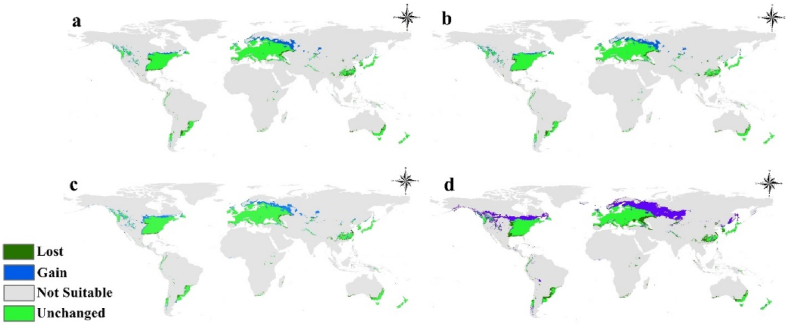
Future distribution calibration maps for *Pseudomonas syringae* worldwide under two representative concentration pathways: a. 2050 for 2.6; b. 2050 for 8.5; c. 2070 for 2.6; and d. 2070 for 8.5 were made.

The study conducted a comprehensive evaluation of future scenarios in relation to climate change, specifically focusing on the habitat suitability of *Pseudomonas syringae*. Four scenarios were considered, including two Representative Concentration Pathways (RCPs) for the years 2050 and 2070 (Fig. 4a–d). This analysis revealed a notable increase in habitat suitability for *Pseudomonas syringae* across various regions worldwide, with particular emphasis on North Europe, Central North America, and Asia. Of particular concern is the RCP 8.5 scenario in 2070, which presents the most alarming projections regarding the suitability of habitats for *Pseudomonas syringae*. This scenario indicates a significant escalation in habitat suitability, particularly in North Europe and Central Asia. These findings underscore the potential consequences of climate change on the distribution and spread of this pathogenic bacterium (Fig. 4a–d). The calibration maps utilized in this study provide explicit visual representations of the observed trends. These maps serve as valuable tools for understanding the spatial patterns of habitat suitability changes and further support the study's conclusions (Fig. 5a–d).

## 4 Discussion

According to the Global Invasive Species Program (GISP), stopping alien species from entering new areas is more practical and cost-effective than controlling their spread after invasion [30]. Therefore, for improved planning and decision-making of control and management actions, it is highly essential to understand the effects of climate change on the possible distribution and range shifts of these bacterial species [31]. We showed that the overall potential suitability for *Pseudomonas syringae* will expand in the future under RCP 8.5 in 2070 scenarios relative to the current climate and other future conditions using all available global occurrence data and accurate species-responsive environmental predictors.

This study highlights the effectiveness of MaxEnt modeling in predicting the distribution of *Pseudomonas syringae*. This method is widely utilized to examine how climate change influences species distribution across various biota [[32], [33], [34]]. The findings suggest that the bacterium may extend its range toward the poles due to rising temperatures, potentially invading new areas and spreading diseases to additional host plants. Furthermore, the annual mean temperature (bio_1) emerges as the primary factor driving the distribution of *Pseudomonas syringae* within a favorable range. Understanding these shifts in habitat suitability can inform the development of targeted strategies for disease management and prevention in specific regions [35].

Our findings are consistent with previous studies that have used similar approaches to study the potential impacts of climate change on the distribution of other microorganisms. For example, a study by Ref. [36] used MaxEnt modeling to predict the distribution of the fungal pathogen *Fusarium oxysporum* under future climate scenarios. The study found that the fungus was likely to expand its range towards the poles, potentially leading to invading new areas and spreading disease to new host plants. Similarly, a study by Saputra et al. [37] used MaxEnt modeling to predict the distribution of the bacterial pathogen *Xanthomonas* spp. under changing climate conditions. The study found that the bacterium was likely to expand its range towards the poles, potentially leading to the invasion of new areas and the spread of disease to new host plants. The same results have been applied to medically necessary microorganisms [32]. Recently, the study of species distribution modeling and climate change impact of the pathogenic bacterium *Xanthomonas oryzae* showed how modeling the future could help mitigate the impact and predict what we will face in the future [38].

The results of these studies, including the present study, suggest that climate change may significantly impact the distribution of microorganisms, including plant pathogens, and may lead to the invasion of new areas and the spread of disease to new host plants. This has important implications for agriculture, forestry, and other industries that rely on the health of plants. However, it is essential to note that the predictions made by MaxEnt modeling are based on several assumptions, including that the climate variables used in the model are the most critical factors influencing the distribution of the microorganism and that the relationship between the climate variables and the distribution of the microorganism remains constant over time. Therefore, it is essential to validate the predictions made by MaxEnt modeling with additional data and research. The implications of these results for scientific publication are significant. They shed light on the potential impacts of climate change on global agriculture and food security, emphasizing the urgent need for proactive measures to mitigate the associated risks posed by *Pseudomonas syringae* and other pathogens. Furthermore, these findings underscore the importance of ongoing monitoring efforts and adaptive strategies to effectively address the anticipated changes in the future.

This study focused on assessing the risk of invasion of the pathogenic bacterium *Pseudomonas syringae* under changing climate conditions. By utilizing GIS and MaxEnt modeling techniques, the researchers predicted the current and future distribution of *P. syringae* and identified the climatological factors influencing its dynamics. The study's findings revealed that *P. syringae*, a significant threat to crops worldwide, is expected to expand its distribution in the future, with areas of high suitability shifting towards the poles. The vital climatic factors driving *P. syringae*'s occurrence were temperature, precipitation, and humidity. The study highlighted the complex and multifaceted relationship between *P. syringae* and climate, emphasizing the role of climate in shaping the biology, ecology, and epidemiology of this plant pathogenic bacterium. The research outcomes provide valuable insights for developing targeted disease management strategies. With climate change, the incidence of bacterial diseases in crops is potentially increasing, and proactive measures are necessary to mitigate such effects. Integrating species occurrence records, climate data, and modeling techniques offers a spatially explicit representation of climatic conditions, enabling the identification of climate patterns associated with disease outbreaks and the characterization of climate suitability for bacterial growth and pathogenicity.

A significant constraint of this study is the exclusive dependence on climatic conditions for modeling the dispersion of *P. syringae*. This is due to the need to compare between current and future situations as a result of climate change. Temperature, precipitation, and humidity have a crucial role in shaping the bacterium's ecology and epidemiology. However, there are additional elements that might have a substantial impact on its geographic distribution and prevalence. Factors such as the distribution and management of host crops, soil conditions, and human activities, including agricultural practices and pathogen transmission, are all important in determining the overall distribution of *P. syringae*. Future research should strive to include these supplementary variables in the modeling framework to offer a more comprehensive evaluation of the existing and future distribution of the bacteria, especially on the local scale. Enhanced comprehension of the intricate connections between climate change, crop production, land use, and human effects can be achieved by incorporating integrating elements. This will provide a more comprehensive knowledge of how these factors contribute to the spread of this significant plant pathogen, taking into account both environmental and socioeconomic causes. To improve the usefulness of the modeling results in guiding disease management methods in response to climate change, it may be essential to address these constraints.

## 5 Conclusion

This study employed (GIS) and MaxEnt modeling to evaluate the present and projected worldwide spread of the plant pathogenic bacteria *Pseudomonas syringae* in response to climate change scenarios. The MaxEnt model had outstanding predictive capability, with an AUC score of 0.92, which suggests that this strategy is very suitable for predicting the geographic distribution of this economically significant bacteria. The research findings offer valuable insights into the main climatic parameters that affect the occurrence and dissemination of *P. syringae*. The adaptability of different places for the growth and proliferation of this bacterium is mainly determined by temperature, precipitation, and humidity. According to the model estimates, climate change is expected to increase the geographic range of *P. syringae*. This means that the places with the most suitable climate conditions for the bacteria will move towards higher latitudes in the next several decades. These findings emphasize the substantial influence that climate change could potentially exert on the frequency and intensity of bacterial infections in crop plants. As the appropriate environment for *P. syringae* expands, the likelihood of infection and reduction in crop production in significant farming areas is anticipated to rise. This highlights the urgent requirement for proactive disease management measures that consider the changing range of this pathogen. The findings of this study can provide valuable guidance for the creation of focused surveillance, timely warning systems, and flexible disease management strategies. By predicting the possible changes in the geographic range of *P. syringae*, individuals involved in the agricultural industry can more effectively plan and execute specific strategies to protect the world food supply from the increasing danger of bacterial plant diseases in a changing environment.

**Declaration of competing interests:** The authors declare that they have no known competing financial interests or personal relationships that could have appeared to influence the work reported in this paper.

## CRediT authorship contribution statement

**Sameh M.H. Khalaf:** Writing – original draft, Validation, Supervision, Methodology, Investigation, Formal analysis, Data curation, Conceptualization. **Monerah S.M. Alqahtani:** Writing – review & editing, Writing – original draft, Methodology, Investigation. **Mohamed R.M. Ali:** Validation, Software, Investigation. **Ibrahim T.I. Abdelalim:** Writing – review & editing, Writing – original draft, Methodology. **Mohamed S. Hodhod:** Writing – original draft, Validation, Supervision, Methodology, Data curation, Conceptualization.

## Ethical statement

Not applicable. This work does not require an ethical statement as it concerns only the analysis of data.

## Data availability

All data, including the manuscript. Occurrence records are available for download from GIBF [20], and the climatological data are freely available from World Clime (https://www.worldclim.org/)

## Declaration of competing interest

The authors declare that they have no known competing financial interests or personal relationships that could have appeared to influence the work reported in this paper.
