# Supplementary material for: Using MaxEnt modeling to analyze climate change impacts on Pseudomonas syringae van Hall, 1904 distribution on the global scale
Source: Heliyon. 2024 Dec 7;10(24):e41017. doi: 10.1016/j.heliyon.2024.e41017 (PMC11696772; doi:10.1016/j.heliyon.2024.e41017)
Supplement: Multimedia component 1 [file mmc1.pdf]

Bioclimatic Factors are coded as follows:

BIO1 = Annual Mean Temperature
BIO2 = Mean Diurnal Range (Mean of monthly (max temp - min temp))
BIO3 = Isothermality (BIO2/BIO7) (×100)
BIO4 = Temperature Seasonality (standard deviation ×100)
BIO5 = Max Temperature of Warmest Month
BIO6 = Min Temperature of Coldest Month
BIO7 = Temperature Annual Range (BIO5-BIO6)
BIO8 = Mean Temperature of Wettest Quarter
BIO9 = Mean Temperature of Driest Quarter
BIO10 = Mean Temperature of Warmest Quarter
BIO11 = Mean Temperature of Coldest Quarter
BIO12 = Annual Precipitation
BIO13 = Precipitation of Wettest Month
BIO14 = Precipitation of Driest Month
BIO15 = Precipitation Seasonality (Coefficient of Variation)
BIO16 = Precipitation of Wettest Quarter
BIO17 = Precipitation of Driest Quarter
BIO18 = Precipitation of Warmest Quarter
BIO19 = Precipitation of Coldest Quarter
